# Uptake of biosimilar trastuzumab in Denmark compared with other European countries: a comparative study and discussion of factors influencing implementation and uptake of biosimilars

**DOI:** 10.1007/s00228-021-03155-4

**Published:** 2021-05-18

**Authors:** Samuel Azuz, Max Newton, Dorthe Bartels, Birgitte Klindt Poulsen

**Affiliations:** 1 Department of Clinical Pharmacology, Aalborg University Hospital, Mølleparkvej 4 8a, 9000 Aalborg, Denmark; 2 Global Supplier & Association Relations, IQVIA, London, UK; 3 Amgros - The Regions’ Pharmaceutical Organisation, Copenhagen, Denmark; 4 Department of Clinical Pharmacology, Aalborg University Hospital, Aalborg, Denmark

**Keywords:** Biological drugs, Biosimilars, Non-medical shift, Oncologics, Pharmacoeconomics

## Abstract

**Purpose:**

The aim of this study was to describe the implementation and uptake of biosimilar trastuzumab in Denmark compared with other European countries.

**Methods:**

European data for usage of trastuzumab was supplied by IQVIA™, using the MIDAS® dataset. A comparison was performed based on market share estimated in sales volume. A separate comparison was undertaken between countries with a full two-fold switch between different biosimilars. Data was collected spanning the time from first registered sales of biosimilar trastuzumab until the 1st quarter of 2020.

**Results:**

Denmark had the fastest and most thorough uptake of biosimilar trastuzumab compared with other EU countries. After 3 months, the market share of biosimilar trastuzumab had increased to 90% while the second fastest country had a 50% market share after 3 months. Only two other countries had undergone a full second switch between biosimilars, Hungary and Norway. All of the three countries made near complete switches between biosimilars while only Denmark had reduced the use of biooriginator below 10%.

**Conclusion:**

The implementation of biosimilar trastuzumab in Denmark was rapid and achieved high overall uptake compared with other EU countries. The switch from one biosimilar to another was also achieved quickly and thoroughly. We believe that the rapid dissemination of information and involvement of all stakeholders — administrators, pharmacies, prescribers, nurses, and patients — constitute the backbone of the Danish success. A similar strategy is recommend for biosimilar implementation in other countries.

## Introduction

Biological medicines (biologics) encompass a large range of different molecules produced via biological processes. Measured in sales value the monoclonal antibodies (MABs) comprising the most important group of biologics [1].

The use of monoclonal antibodies as biological treatment has not only revolutionized the care for patients in several fields, but also substantially increased the financial burden for healthcare systems. Biosimilars have been hailed as a possible relief of this burden. The switch from original biologics (biooriginators) to biosimilars, however, has varied substantially between countries [2, 3].

The swift uptake of anti-inflammatory biosimilars in a Danish setting has been described earlier [4, 5]. For oncologic biosimilars, concerns regarding efficacy and safety remain a challenge to swift implementation in some settings, even though no studies have found biosimilar trastuzumab inferior to the biooriginator [6, 7].

Denmark is a relatively small country in the European Union (EU) with a population of 5.8 million. The treatment with monoclonal antibodies is almost exclusively administered via hospitals. Treatment is given free of charge for the patient, and the administering departments are reimbursed via the regional budget, which is tax-funded.

Medicines for hospital use are acquired via national tenders. The tenders are organized by the central medicines’ supplier AMGROS which supplies hospitals in the five Danish regions with approximately 99% of medicines used in hospitals including outpatient clinics.

**Fig. 1:**
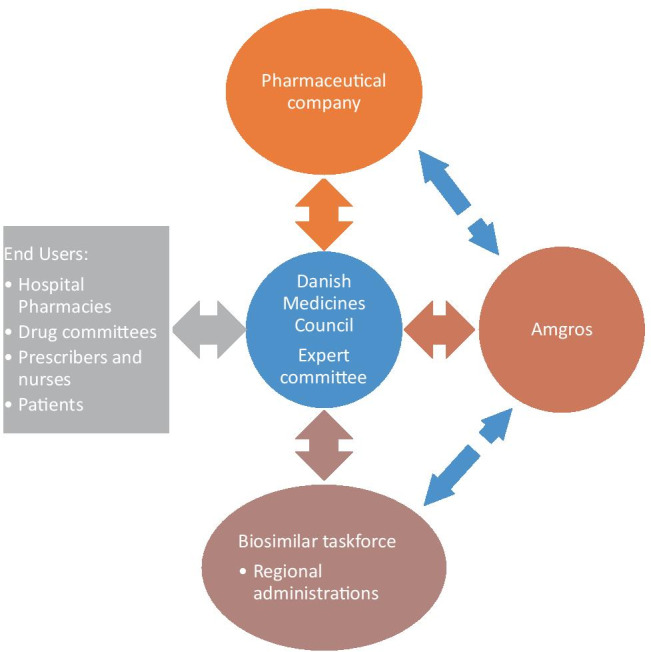
Diagram illustrating the implementation process for biosimilars used in hospitals in Denmark

The Danish Medicines Council (DMC) has a central role in evaluating and implementing biosimilars (Fig. 1). An assessment of the efficacy and safety of the treatment is performed by an expert committee including representative physicians from each region. The differences in expenditures between the biooriginator and biosimilar are analysed, including the differences in workload between formulations/devices—e.g. longer infusion time associated with intravenous formulation (IV) compared to subcutaneous (SC). If the biosimilar is found efficacious, safe and enables significant financial savings, a national guideline is created by the DMC recommending the biosimilar over the biooriginator. A biosimilar task force with representatives from the Danish Regions, the DMC, and AMGROS carefully plans the implementation process [8, 9].

Preparations by the task force involve all stakeholders and commence as soon as a lower-cost alternative has been secured. Information is sent to regional hospital pharmacies to prepare for the implementation and to plan depletion of biooriginator stocks. Prescribing departments, physicians, and patients in current treatment are also thoroughly informed of the incoming switch.

The DMC has now evaluated four biosimilars, and the council’s predecessor The Council for the Use of Expensive Hospital Medicines (RADS) evaluated two further biosimilars. All evaluations have recommended a full implementation to the entire patient population including current and former users of the biooriginator. The Medicines Council has clearly stated that the outset for evaluating biosimilars is that they are equal in terms of safety and quality compared to biooriginators [10].

In Denmark, the first implementation of biosimilar trastuzumab started in the summer of 2018 with a switch to a second biosimilar in the beginning of 2020. However, this implementation has not yet been systematically described.

The aim of this study was to describe the implementation and uptake of biosimilar trastuzumab in Denmark compared with other European countries. A separate analysis was also undertaken with countries which had also undergone a full second switch from one biosimilar to another.

## Methods

European sales volumes were retrieved from MIDAS®, an IQVIA™ analytics platform which tracks the sales of medicines globally on multiple levels ranging from the manufactories all the way to patients. The data from all participating companies is audited annually by IQVIA™. The precision of sales registered versus actual sales confirmed by the audit is expressed as the bias. A precision index is calculated on the basis of the percentage of audited datasets which are inside the overall bias ± 22.5%. The precision index for Europe in 2019 was 92.5% [11].

Data were included for all countries with recorded sales of trastuzumab (18 countries), sales were captured for both hospital, and retail settings in all markets analysed. Treatment days are the measure shown throughout; this is normally based on the defined daily dose (DDD) which is the assumed average maintenance dose per day for a drug used for its main indication in adults. The World Health Organisation (WHO) does not provide DDDs for products in this class, because they do not have sufficient data to calculate DDDs for medicines that are dosed dependent on the body weight of the patient, so average doses were calculated using IQVIA Oncology Dynamics data real-world data on physician prescribing (December 2017) on projected numbers of patients in EU5 (France, Germany, Italy, Spain, UK), accounting for the dosing and length of the treatment cycle. To calculate treatment days, the methodology is derived from the total mass of the product consumed (international product strength × counting units) divided by the average dose above to determine the number of patient days for these products.

Available data covered the entry of biosimilar trastuzumab on the European market in 2018 until the 1st quarter of 2020.

Since biosimilar trastuzumab is currently only available as IV formulation while the biooriginator is also available as SC formulation, which provides easier administration, it was assumed that some countries had decided that the potential savings of switching to IV biosimilar did not outweigh the burden of not using SC biooriginator. Therefore, a cut-off value of > 50% SC formulation usage was chosen as representing a limited implementation of biosimilar use, and a different way of leveraging the benefit of biosimilar competition, and therefore not compared directly to Denmark.

A separate comparison was performed between countries with a full two-fold switch between different biosimilars. Since most countries do not use national tenders, where only one biosimilar dominates the market, there were very few comparable countries with Denmark. The countries found to have undergone a near complete switch between biosimilars were Norway and Hungary.

## Results

**Fig. 2:**
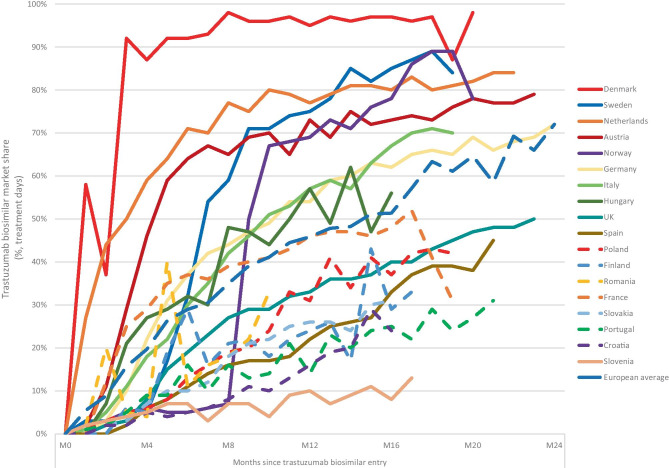
Market share of biosimilar trastuzumab for several EU countries over time. M0 is normalised to the date of first sales in the market recorded in MIDAS®. *Croatia, Finland, France, Portugal, Romania, and Slovakia are shown in stippled lines due to primary use (> 50% market share in 2020) of SC biooriginator formulation. Source: IQVIA MIDAS dataset, Q1 2020 MAT using treatment days (TD)

Denmark had the fastest and most thorough uptake of biosimilar trastuzumab compared with other EU countries (Fig. 2). After 3 months, the market share of biosimilar trastuzumab had increased to 90% while the second fastest Netherlands had a 50% market share. In the long run, Denmark was also the only country to cross the 90% market share boundary.

**Fig. 3:**
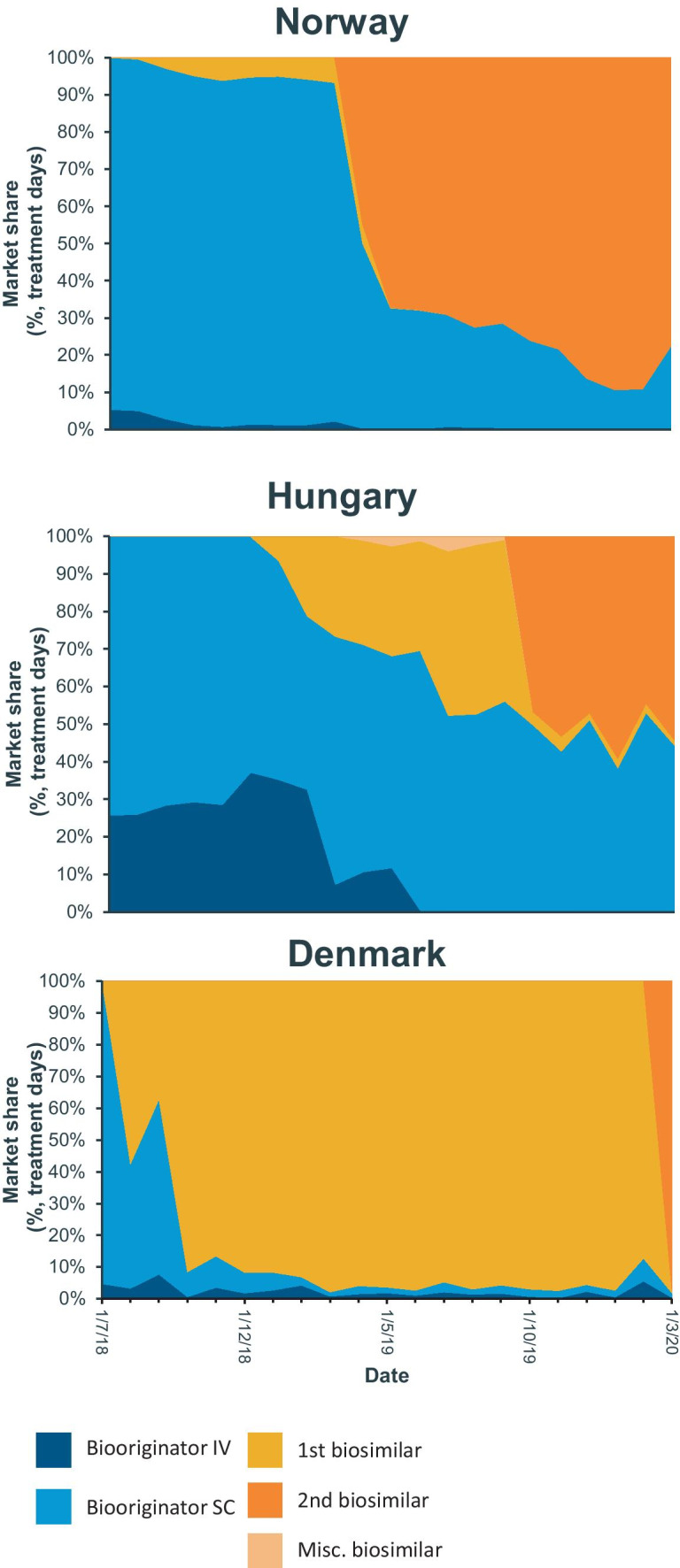
Market share distribution for Norway, Hungary, and Denmark, where a shift from one biosimilar to another had taken place at the time of writing. Source: IQVIA MIDAS dataset, Q1 2020 MAT using treatment days (TD)

When making a detailed comparison with Norway and Hungary, a clear difference was seen in usage of SC versus IV trastuzumab (Fig. 3). At the outset, only Hungary had a significant proportion of IV use at 25% while Norway and Denmark almost exclusively used SC formulation. Within a year, Denmark almost entirely replaced SC with IV biosimilar trastuzumab while Norway and Hungary only partially managed to replace SC biooriginator with an IV biosimilar alternative. Norway kept primarily using SC biooriginator until the second biosimilar switch 8 months after the arrival of the first biosimilar. After the second switch, Norway reduced its use of biooriginator below 20% of the market share. Both Hungary and Denmark made a complete switch from one biosimilar to another; however, the use of SC biooriginator remained at around 50% in Hungary.

## Discussion`

### Factors influencing the implementation and uptake of biosimilars

The most important factor associated with uptake biosimilars is price competitiveness. Several cases of biooriginators winning tenders based on offering the lowest price have been described [12]. This probably also explains why Norway continued to primarily recommend and use biooriginator trastuzumab when biosimilars first became available and switched to primarily using a second biosimilar later [13].

Besides price competitive biooriginators, several challenges to the implementation of biosimilars have been described [14]. Barring the lag time of market entry, due to the resource and time-consuming authorization process via EMA [15] and the more complex manufacturing methods, which can be subject to patents, three factors were identified which can slow and limit the implementation of biosimilars which need to be addressed. These are the following:

1. Patented formulations and indications
2. Market supply and tenders
3. Adoption by subscribers

#### Patents

Although secondary patents may delay the use of biosimilars for some indications—e.g. biosimilar rituximab could initially only be used for non-Hodgkin lymphoma due to secondary patents on other indications—they can also delay the uptake if they are linked to the route of administration [16]. Patents on improved formulations such as subcutaneous administration may force clinical personnel to change work routines when switching to biosimilars. This in turn increases the required cost savings of biosimilars to offset the potential cost of implementation. Furthermore, it requires diligent analysis of workflow and often pragmatic allocation of resources to off-set the more time-consuming IV treatment. In Denmark, several cases of longer opening hours at outpatient clinics, hiring of more nurses and purchasing of transfusion chairs have been necessary. It could be assumed that the allocation of resources to such pragmatic solutions requires support on an administrative level beyond the local clinical personnel. In a study, describing the implementation of biosimilar adalimumab in the EU countries, Scotland was the only country that reported having planned for additional staffing [17].

Evaluating to what extent trastuzumab is used in an ambulatory care setting in different countries is beyond the scope of this study, but if administration is primarily performed in such a setting, it would heavily favour SC formulations.

Some pharmaceutical companies have chosen to compete mainly with improved formulations of biooriginators against biosimilars, which in part, may explain why several European countries primarily (> 50% market share) used the SC formulation of trastuzumab [12].

#### Market and tenders

The availability of biosimilars may vary among locations. It is entirely the prerogative of the industry to decide where to actively market its products. Some countries in the EU have a very limited use of biooriginator to begin with, and some markets have been unable to increase availability even post-biosimilar entry (e.g. in the anti-TNF class in Poland, Romania, and Italy) [12]. Maximizing the potential market by recommending the treatment to the entire patient population (new, previous, and current users of biooriginators) with large tenders combined with a swift and thorough implementation could incentivize several manufactures to bid on tenders, thereby increasing competition and drive prices down.

In Denmark, tenders are national and usually with only one winner based on price alone. This, combined with a swift uptake, puts an enormous strain on the supplier with an expected capacity to deliver from day one. It also makes bidding on tenders in Denmark somewhat risky, as a lost tender necessitates reallocation of the entire supply or scrapping it if this is impossible. However, in some cases, where one supplier is unable to guarantee sufficient supply, more winners can be chosen to supply different regions of Denmark.

Tendering practices vary as widely as the structure of healthcare systems across the EU [18]. It seems that national tenders correlate with faster and higher uptake [19]. However, countries without national tenders can also achieve high uptake of biosimilars as shown by Sweden that uses regional tenders and achieved > 70% market share for biosimilar trastuzumab within a year. The importance of tendering practices with regard to the long-term sustainability of the biosimilar market versus short-term lowering of prices is, however, an interesting area of research and debate. Denmark alone was able to achieve a market share above 90% that can partly be attributed to the tendering process. It does, however, not explain why the market share increased markedly quicker compared to other countries.

#### Adoption by prescribers

Probably the largest factor delaying implementation of biosimilars is actual use by prescribers. Willingness to prescribe biosimilars may be limited due to several reasons:

- Prescriber and/or patient insecurity concerning efficacy and safety
- Conservative prescribing patterns
- Reduced practicality — subcutaneous vs intravenous administration
- Lack of financial motivation — reimbursement policies.

Survey studies have found that prescribers are generally cautious towards using biosimilars and that their knowledge about biosimilars is lacking. All surveys point towards a need for more education about biosimilars. [20–22] Studies describing how to counter the prescribers limited use of biosimilars are few — to our knowledge, only two studies exists that describes the process in detail [23, 24].

In countries with historically low usage, prescribers with limited experience of biologic medicines remain conservative in their prescribing patterns, keeping patients on other treatments before moving across to the bioorigionator or biosimilar. These patterns of prescribing are suggested to be a part of the delay in many markets where prices have fallen after biosimilar entry, but volume remained low.

This underscores the importance of the dissemination of information to all affected parties during an implementation process by the DMC. All these factors need to be managed in order to ensure that implementation is not prolonged or outright hampered. We believe that the Danish solution with a centralized task force which rapidly informs and involves all stakeholder constitutes a cornerstone in the fast and thorough uptake seen in Denmark. Furthermore, the more ingrained an implementation process becomes through previous experiences, the more efficient and effortless it becomes. A well planned implementation process with timely involvement of all stakeholders should, probably, be seen as a gold-standard approach to biosimilar implementation.

## Conclusion

The implementation of biosimilar trastuzumab in Denmark was rapid and achieved high overall uptake compared with other EU countries. The switch from one biosimilar to another was also achieved quickly and thoroughly.

## Recommendations

The varied landscape of European healthcare systems limits the capability to emulate the Danish approach to biosimilar implementation. The single-winner tender approach is not the sole reason for the success of Danish biosimilar implementation or the savings achieved. Larger EU markets using this same approach, however, would have a negative impact on the sustainability of the biosimilars market, reducing the long-term potential for future savings.

Multi-winner tenders can achieve equivalent levels of savings. The speed of the Danish implementation is remarkably unique and must be attributed the thorough preparations for the implementation. Therefore, there are many learnings that can be used in any market in Europe and have now been shown to be crucial. First of all, implementation requires careful planning well before the arrival of the new biosimilar treatment. We believe that the rapid dissemination of information and involvement of all stakeholders — administrators, pharmacies, prescribers, nurses, and patients — constitute the backbone of the Danish success and strongly recommend a similar approach in other countries.

## Support

The creation of this article was supported with a research grant to the Department of Clinical Pharmacology, Aalborg University Hospital by MSD Nordic.
